# The relationship between impulsive behaviors and adult attention-deficit/hyperactivity disorder symptoms in bipolar disorder

**DOI:** 10.3389/fpsyt.2026.1916550

**Published:** 2026-10-09

**Authors:** Tülay Sati Kirkan, Nazan Dolapoglu

**Affiliations:** 1 Department of Psychiatry, Faculty of Medicine, Bandırma Onyedi Eylül University, Bandırma, Türkiye; 2 Department of Psychiatry, Faculty of Medicine, Balıkesir University, Balıkesir, Türkiye

**Keywords:** ASRS, attention-deficit/hyperactivity disorder, Barratt Impulsiveness Scale, bipolar disorder, euthymia, impulsivity

## Abstract

**Objective:**

This study aimed to examine the relationship between impulsivity and adult Attention-Deficit/Hyperactivity Disorder (ADHD) symptoms in euthymic patients with Bipolar Disorder (BD) Type I.

**Methods:**

One hundred and eleven outpatients diagnosed with BD Type I according to DSM-5 criteria, scoring ≤7 on the Hamilton Depression Rating Scale (HAM-D) and ≤5 on the Young Mania Rating Scale (YMRS), and in remission for at least two months were included. Impulsivity was assessed with the Barratt Impulsiveness Scale-11 (BIS-11) and ADHD symptoms with the Adult ADHD Self-Report Scale (ASRS). Associations were evaluated with Spearman correlation analysis with Benjamini–Hochberg false discovery rate (FDR) correction, and hierarchical multivariable linear regression. Sensitivity analyses addressed item-content overlap, influential observations, and medication effects.

**Results:**

A strong positive correlation was found between BIS-11 total and ASRS total scores (ρ=0.722; q<0.001); the ASRS Inattention and Hyperactivity-Impulsivity subscales were themselves moderately intercorrelated (ρ=0.600; p<0.001). Only 18.9% of patients scored ≥72 on the BIS-11 (clinically significant range) and 28.8% screened positive on ASRS Part A. In the final regression model, ASRS total score was the strongest independent predictor of BIS-11 total score (B = 0.734; β=0.676; p<0.001), together with Young Mania Rating Scale score (β=0.170; p=0.018; R^2^=0.573), an association that strengthened after excluding influential observations and after dichotomization (both p<0.01); variance inflation factors (1.04–2.61) indicated no multicollinearity. Both ASRS subscales remained independent predictors in subscale analysis (Hyperactivity-Impulsivity β=0.436; Inattention β=0.317; both p<0.001). The association persisted after removing shared item content (ρ=0.605; p<0.001), excluding influential observations, and adjusting for treatment regimen.

**Conclusion:**

In euthymic BD Type I patients, self-reported impulsivity showed a strong, independent association with self-reported adult ADHD symptom burden, particularly the hyperactivity-impulsivity dimension, robust across sensitivity analyses. Because ADHD symptoms were assessed only by self-report screening, without a structured diagnostic interview, and because the design is cross-sectional, these findings reflect a symptom-level, not diagnostic, association and do not establish causality. They nonetheless support considering ADHD symptoms in the routine clinical evaluation of euthymic BD Type I patients.

## Introduction

Bipolar disorder (BD) is a psychiatric disorder characterized by a lifetime prevalence of approximately 1%, high rates of recurrence, comorbidity, and functional impairment (1, 2). Current literature indicates that BD is not limited to acute mood episodes; rather, neurocognitive deficits persist even during remission (euthymic) periods (3). Among these deficits, impairments particularly affecting attention, executive functions, memory, and processing speed are considered trait characteristics of the disorder (4).

Impulsivity, which plays a central role in the pathophysiology of BD, is defined as both a state-related and trait-related component throughout the course of the illness. Impulsivity encompasses a multidimensional construct involving acting without forethought, inability to delay gratification, and difficulty sustaining attention (5, 6). Previous studies have demonstrated that impulsive traits measured by self-report scales (such as the BIS-11) remain significantly elevated in patients with BD compared with healthy controls, regardless of illness phase (7). Furthermore, high levels of impulsivity have been reported to increase the risk of suicide attempts among patients with BD (8).

The clinical similarities and high comorbidity rates between BD and Attention-Deficit/Hyperactivity Disorder (ADHD) pose a significant challenge in diagnostic processes. The prevalence of comorbid ADHD among adults with BD has been reported to reach 17–20% in the literature (9, 10). Both disorders share common symptom clusters, including emotional dysregulation, impulsivity, and difficulties in cognitive control (11). In particular, the chronic nature of ADHD, persisting from childhood onward, resembles the residual symptoms observed during inter-episode periods of BD, thereby complicating differential diagnosis (12).

Neuropsychological studies have demonstrated that both BD and ADHD patients exhibit similar deficits on measures of executive functioning; however, individuals with BD may experience more pronounced difficulties in the domain of verbal memory (11). Whether cognitive functioning is more severely impaired in individuals with comorbid BD and ADHD compared with those with BD alone remains an ongoing area of investigation (4).

The present study aims to systematically examine the relationship between impulsive behavioral patterns that persist during euthymic periods and adult ADHD symptoms in individuals diagnosed with BD. Given the shared symptom domains and the neurobiological overlap between BD and ADHD described above, we hypothesized that impulsivity severity would be significantly and independently associated with ADHD symptom burden—particularly the hyperactivity-impulsivity dimension—even after adjustment for demographic, clinical, and residual mood-symptom variables. Clarifying the shared dimension of impulsivity between these two conditions is of clinical relevance for follow-up and treatment planning, given that impulsivity has separately been linked to adverse outcomes, including suicide risk, in the broader BD literature; the present study does not itself test the relationship between ADHD symptoms and suicide risk.

## Methods

### Study design and sample

This cross-sectional and descriptive study investigated the relationship between impulsivity and adult ADHD symptoms in patients diagnosed with BD. The study sample consisted of volunteer patients diagnosed with Bipolar Disorder Type I according to the DSM-5 (13) diagnostic criteria, who attended the psychiatry outpatient clinic of the Faculty of Medicine, Bandırma Onyedi Eylül University, between March 15, 2023 and March 15, 2024. Diagnoses were confirmed by a specialist psychiatrist using the Structured Clinical Interview for DSM-5 (SCID-5); no participant received a first-time BD diagnosis in the context of this study, as all had a pre-existing diagnosis that was reconfirmed at enrollment. Participants were recruited from consecutive attenders at the study clinics during the study period who met the inclusion and exclusion criteria below. Candidates were followed for a two-month period to verify sustained euthymia (HAM-D ≤7, YMRS ≤5) before the study scales were administered, rather than being assessed at a single cross-sectional timepoint.

Inclusion criteria were as follows (1): age between 18 and 65 years (2); completion of at least primary school education (3); HAM-D score ≤7 (4); YMRS score ≤5; and (5) remission for at least two months. Exclusion criteria were (1): the presence of a neurological disorder or organic mental disorder severe enough to interfere with the interview (2); being in a depressive, manic, hypomanic, or mixed episode (3); having received electroconvulsive therapy (ECT) within the previous six months; and (4) active substance use disorder.

All participants were informed about the study and provided written informed consent prior to participation. Ethical approval was obtained from the Non-Interventional Clinical Research Ethics Committee of Bandırma Onyedi Eylül University (Approval No: 2023-28), and the study was conducted in accordance with the principles of the Declaration of Helsinki.

### Data collection instruments

#### Sociodemographic and clinical information form

This form, developed by the researchers, included information regarding sex, date of birth, years of education, marital status, occupation, age at illness onset, duration of illness, total number of episodes (mania, hypomania, depression, mixed episodes), current duration of euthymia, psychiatric medications and duration of use, number of hospitalizations, number of suicide attempts, comorbid physical illnesses, family history of psychiatric and physical illnesses, body weight, height, body mass index (BMI), and alcohol and tobacco use. Total number of episodes, number of hospitalizations, and number of suicide attempts were obtained from patients’ electronic and physical medical records and cross-verified during the clinical interview against history obtained from the patient and, where available, a first-degree relative.

#### Adult attention-deficit/hyperactivity disorder self-report scale

The ASRS is an 18-item self-report screening scale developed by Kessler and colleagues under the coordination of the World Health Organization (14). It consists of two subscales: inattention and hyperactivity-impulsivity. Each subscale contains nine items, and responses are scored on a scale ranging from 0 to 4 (0 = never, 4 = very often). The Turkish validity and reliability study of the scale was conducted by Doğan et al.; the test reliability coefficients of the Turkish version ranged between 0.73 and 0.89, and the Cronbach’s alpha coefficient was reported as 0.88 (15).

#### Hamilton depression rating scale

This clinician-administered scale, developed by Max Hamilton, assesses the severity of depressive symptoms (16). It consists of 17 items, each scored on a scale ranging from 0 to 4. The Turkish validity and reliability study was conducted by Akdemir et al. in 1996; the Cronbach’s alpha coefficient was reported as 0.75, the Spearman–Brown coefficient as 0.76, and the inter-rater reliability coefficient ranged between 0.86 and 0.98 (17).

#### Young mania rating scale

The YMRS is an 11-item clinician-administered scale developed by Young and colleagues in 1978 to assess the severity of manic symptoms (18). The Turkish validity and reliability study was conducted by Karadağ et al.; the internal consistency coefficient was reported as 0.79, and item-total score correlations ranged between 0.407 and 0.847 (19).

#### Barratt impulsiveness scale-11

The BIS-11 is a 30-item self-report scale used to assess impulsivity (20). Items are scored on a scale ranging from 1 to 4 (1 = never/rarely, 4 = almost always/always); items 1, 7, 8, 9, 10, 12, 13, 15, 20, 29, and 30 are reverse-scored prior to summation. The scale comprises three subscales: Attention (inattention and cognitive instability), Motor (motor impulsivity and perseverance deficits), and Non-Planning (lack of self-control and intolerance of cognitive complexity). The Turkish validity and reliability study of the scale was conducted by Güleç et al. (21).

### Data collection procedure

Data were collected through face-to-face interviews and self-report questionnaires.

### Statistical analysis

Statistical analyses were performed using IBM SPSS Statistics for macOS, version 30.0 (IBM Corp., Armonk, NY, USA). The normality of continuous variables was assessed using the Shapiro–Wilk test, examination of skewness and kurtosis values within the range of ±2, and visual inspection of histogram plots. Continuous variables were presented as mean ± standard deviation (SD) or median (interquartile range, IQR), depending on their distributional characteristics, whereas categorical variables were expressed as frequency and percentage [n (%)].

Associations among psychometric scales were evaluated using Spearman correlation analysis. Because the correlation matrix comprised multiple pairwise comparisons, the Benjamini–Hochberg procedure was applied to control the false discovery rate (FDR), and FDR-corrected p-values are reported as q-values, with q<0.05 considered statistically significant. Hierarchical (blockwise) multivariable linear regression analysis was performed to identify factors associated with the BIS-11 total score. In the first block, the ASRS total score (and ASRS subscale scores in the sub-analysis) was entered into the model. Demographic variables (age, sex, and years of education) were entered in the second block, and clinical variables (duration of illness, total number of episodes, HAM-D score, YMRS score, and smoking status) were entered in the third block. Regression assumptions were examined with respect to the distribution of residuals, linearity, homoscedasticity, and independence of residuals via residual plots and diagnostic statistics, and no serious violations were identified on visual inspection; multicollinearity was assessed using variance inflation factor (VIF) and tolerance values, and influential observations were identified using Cook’s distance (cases with Cook’s D >4/n were considered influential). To evaluate the possible contribution of item-content overlap between the ASRS and the BIS-11 to the principal association, a sensitivity analysis was performed after removing conceptually and behaviorally overlapping items (ASRS items 1, 2, 4, 5, 8, 11, 12, 15, 16, 17, and 18; BIS-11 items 1, 5, 6, 7, 9, 11, 14, 17, 19, and 28); the resulting reduced scores were re-examined using Spearman correlation and multivariable linear regression adjusted for the same covariates. The sensitivity of the main regression model to influential observations was assessed by repeating the analysis after excluding cases with Cook’s D >4/n. A further sensitivity analysis added treatment regimen (monotherapy vs. combination therapy) and duration of medication use to the main model. The adequacy of the sample size for the regression analyses was evaluated with a *post-hoc* sensitivity power analysis (PASS 11; α=0.05, power=80%). ASRS Part A screening positivity was defined as meeting the recommended threshold on at least four of the six Part A screening items. Model explanatory power was evaluated using R^2^ and adjusted R^2^ values. For all other analyses, a two-tailed p-value <0.05 was considered statistically significant.

## Results

Of the 111 patients included in the study, 56.8% (n=63) were female and 43.2% (n=48) were male. The mean age was 45 ± 12 years, and the mean duration of education was 11 ± 4 years. The mean body weight was 85.1 ± 15.4 kg, mean height 168.2 ± 9.3 cm, and mean BMI 30.2 ± 5.6 kg/m^2^, indicating that the patient group was, on average, within the obesity range. Regarding marital status, 51.4% (n=57) were married, 37.8% (n=42) were single, 9.9% (n=11) were widowed, and 0.9% (n=1) were divorced; the most common occupational group was homemakers (27.9%). Alcohol use was reported by 13.5% (n=15) of participants (median amount among users 3, IQR 1–4) and cigarette smoking by 63.1% (n=70) (median amount among users 1, IQR 1–2). The mean age at illness onset was 28 ± 9 years, and the mean duration of illness was 17 ± 10 years. The median total number of episodes was 8 (IQR 5–15); among patients with a relevant episode history, the median number of manic episodes was 3 (IQR 2–5, n=111), hypomanic episodes 2 (IQR 2–5, n=74), depressive episodes 3.5 (IQR 2–5, n=94), and mixed episodes 2 (IQR 1–3, n=38). The median duration of current euthymia was 2 years (IQR 1–3.5). A history of psychiatric hospitalization was present in 82.0% (n=91) of patients, with a median of 2 hospitalizations (IQR 1–3.5) among those with such a history. A history of suicide attempts was present in 20.7% (n=23) of patients, with a median of 2 attempts (IQR 1–3) among those with a history of attempts. The demographic, clinical, and lifestyle characteristics of the patients are summarized in **Table 1**.

**Table 1:** Distribution of demographic, clinical, and lifestyle characteristics of the patients.

Variables (N = 111)	n (%)	Mean ± SD	Median (IQR)
Sex			
Male	48 (43.2)		
Female	63 (56.8)		
Age (years)		45 ± 12	
Weight (kg)		85.1 ± 15.4	
Height (cm)		168.2 ± 9.3	
BMI (kg/m^2^)		30.2 ± 5.6	
Years of education		11 ± 4	
Marital status			
Single	42 (37.8)		
Married	57 (51.4)		
Divorced	1 (0.9)		
Widowed	11 (9.9)		
Occupation			
Homemaker	31 (27.9)		
Unemployed	16 (14.4)		
Manual worker	26 (23.4)		
Civil servant	18 (16.2)		
Retired	20 (18.0)		
Alcohol use	15 (13.5)		
Amount among users, n=15			3 (1-4)
Cigarette smoking	70 (63.1)		
Amount among users, n=70			1 (1-2)
Age at illness onset (years)		28 ± 9	
Duration of illness (years)		17 ± 10	
Total number of episodes			8 (5-15)
Manic episodes, n=111			3 (2-5)
Hypomanic episodes, n=74			2 (2-5)
Depressive episodes, n=94			3.5 (2-5)
Mixed episodes, n=38			2 (1-3)
Duration of euthymia (years)			2 (1-3.5)
History of hospitalization	91 (82.0)		
Number of hospitalizations, n=91			2 (1-3.5)
History of suicide attempts	23 (20.7)		
Number of suicide attempts, n=23			2 (1-3)

Categorical variables are presented as n (%), whereas continuous variables are presented as mean ± SD or median (IQR) according to their distributional characteristics. BMI, Body Mass Index.

The most commonly used treatment regimen was the combination of a mood stabilizer and an antipsychotic (67.6%). The median duration of medication use was 3 years (IQR 2–5). No comorbid physical illness was present in 68.5% of the patients; among those with comorbid conditions, internal medicine-related disorders (24.3%) were the most common. Regarding family history, 31.5% of the patients reported a family history of psychiatric illness, whereas 55.9% reported a family history of physical illness. Treatment characteristics and comorbidity data are presented in **Table 2**.

**Table 2:** Distribution of treatment characteristics and comorbid conditions.

Variable	n (%)	Median (IQR)
Medications used		
MS + AP	75 (67.6)	
MS alone	7 (6.3)	
AP alone	10 (9.0)	
MS + AD	4 (3.6)	
AP + AD	3 (2.7)	
MS + AP + AD	12 (10.8)	
Duration of medication use (years)		3 (2-5)
No comorbid physical illness	76 (68.5)	
Family history of psychiatric illness	35 (31.5)	
Family history of physical illness	62 (55.9)	

Categorical variables are presented as numbers and percentages [n (%)], and continuous variables are presented as median (interquartile range, IQR). MS, Mood stabilizer; AP, Antipsychotic; AD, Antidepressant.

The mean BIS-11 total score was 58.8 ± 13.6. Mean subscale scores were 14.6 ± 4.9 for Attention, 18.9 ± 5.1 for Motor, and 25.3 ± 5.9 for Non-Planning. The mean ASRS total score was 28.9 ± 12.5, with mean Inattention and Hyperactivity-Impulsivity subscale scores of 16.1 ± 7.3 and 12.8 ± 6.6, respectively. The median HAM-D score was 2 (IQR 0–5), and the median YMRS score was 0 (IQR 0–0.5). Applying the conventional BIS-11 cut-off, 18.9% (n=21) of patients scored ≥72, the range regarded as clinically significant impulsivity; applying the validated ASRS Part A screening algorithm, 28.8% (n=32) of patients screened positive. Psychometric scale scores and screening results are summarized in **Table 3**.

**Table 3:** Distribution of psychometric scale scores.

Variable	n (%)	Mean ± SD	Median (IQR)
BIS-11 Total		58.8 ± 13.6	
BIS-11 Attention		14.6 ± 4.9	
BIS-11 Motor		18.9 ± 5.1	
BIS-11 Non-Planning		25.3 ± 5.9	
HAM-D			2 (0-5)
YMRS			0 (0-0.5)
ASRS Total		28.9 ± 12.5	
ASRS Inattention		16.1 ± 7.3	
ASRS Hyperactivity-Impulsivity		12.8 ± 6.6	
BIS-11 Total ≥72	21 (18.9)		
ASRS Part A screening positive	32 (28.8)		

Continuous variables are presented as mean ± standard deviation or median (interquartile range, IQR) according to their distribution characteristics, while categorical variables are presented as number and percentage [n (%)]. BIS-11, Barratt Impulsiveness Scale-11; HAM-D, Hamilton Depression Rating Scale; YMRS, Young Mania Rating Scale; ASRS, Adult ADHD Self-Report Scale.

Correlation analyses (Spearman) revealed a strong positive correlation between the ASRS total score and the BIS-11 total score (ρ=0.722; q<0.001). The ASRS Hyperactivity-Impulsivity subscale showed a strong correlation with BIS-11 total score (ρ=0.656; q<0.001), as did the ASRS Inattention subscale (ρ=0.631; q<0.001). At the subscale level, the strongest correlation was between the ASRS Hyperactivity-Impulsivity subscale and the BIS-11 Attention subscale (ρ=0.701; q<0.001). The ASRS Inattention and Hyperactivity-Impulsivity subscales were themselves moderately intercorrelated (ρ=0.600; p<0.001). The Young Mania Rating Scale score correlated positively with BIS-11 total, Attention, and Motor scores (ρ=0.293–0.399; q ≤ 0.002), but not with the Non-Planning subscale after FDR correction (ρ=0.191; q=0.056). The Hamilton Depression Rating Scale score was not significantly associated with any BIS-11 score after FDR correction (all q>0.05). The correlation matrix, with both raw and FDR-corrected (q) values, is presented in **Table 4**. For the main regression model (N = 111, nine predictors), a *post-hoc* sensitivity power analysis indicated that, at α=0.05 and 80% power, the minimum detectable overall effect size was Cohen’s f^2^=0.153, well below the effect size observed for the ASRS-BIS-11 association.

**Table 4:** Correlation analysis among psychometric scales.

		BIS-11 total	BIS-11 attention	BIS-11 motor	BIS-11 non-planning
HAM-D	ρ	0.144	0.161	0.004	0.154
	p	0.131	0.092	0.971	0.106
	q	0.138	0.108	0.971	0.118
YMRS	ρ	**0.347**	**0.399**	**0.293**	0.191
	p	**<0.001**	**<0.001**	**0.002**	0.044
	q	**<0.001**	**<0.001**	**0.002**	0.056
ASRS Inattention	ρ	**0.631**	**0.615**	**0.348**	**0.617**
	p	**<0.001**	**<0.001**	**<0.001**	**<0.001**
	q	**<0.001**	**<0.001**	**<0.001**	**<0.001**
ASRS Hyperactivity-Impulsivity	ρ	**0.656**	**0.701**	**0.516**	**0.471**
	p	**<0.001**	**<0.001**	**<0.001**	**<0.001**
	q	**<0.001**	**<0.001**	**<0.001**	**<0.001**
ASRS Total	ρ	**0.722**	**0.733**	**0.473**	**0.623**
	p	**<0.001**	**<0.001**	**<0.001**	**<0.001**
	q	**<0.001**	**<0.001**	**<0.001**	**<0.001**

Spearman correlation analysis was used for all variables. Multiple comparisons were corrected using the Benjamini–Hochberg false discovery rate (FDR) procedure. ρ, Spearman correlation coefficient; p, uncorrected p-value; q, FDR-corrected p-value. Bold, q<0.05. HAM-D, Hamilton Depression Rating Scale; YMRS, Young Mania Rating Scale; ASRS, Adult ADHD Self-Report Scale.

The hierarchical regression analysis evaluating factors associated with the BIS-11 total score is presented in **Table 5**. Model 1, which included only the ASRS total score, was statistically significant and explained 53.4% of the variance (R^2^=0.534; p<0.001). Adding demographic variables (Model 2) did not significantly increase the explained variance (ΔR^2^=0.012; p=0.419), nor did adding clinical variables (Model 3), which raised R^2^ to 0.573 (ΔR^2^=0.026; p=0.301). In the final model, the ASRS total score remained the strongest independent predictor of BIS-11 total score (B = 0.734; β=0.676; p<0.001), and the Young Mania Rating Scale score also emerged as an independent, positive predictor (B = 1.564; β=0.170; p=0.018). Age, sex, years of education, duration of illness, number of episodes, HAM-D score, and smoking status were not independently associated with BIS-11 total score (all p>0.05). Variance inflation factors in the final model ranged from 1.04 to 2.58, indicating no problematic multicollinearity.

**Table 5:** Multivariable linear regression analysis of factors associated with the BIS-11 total score.

Model	Variable	B	β	SE	95% CI	p
1	ASRS Total	**0.794**	**0.731**	**0.071**	**0.653-0.935**	**<0.001**
2	ASRS Total	**0.776**	**0.715**	**0.072**	**0.634-0.919**	**<0.001**
	Age	-0.106	-0.094	0.083	-0.271-0.059	0.206
	Sex	0.057	0.002	2.035	-3.978-4.092	0.978
	Years of education	-0.278	-0.090	0.218	-0.712-0.155	0.205
3	ASRS Total	**0.734**	**0.676**	**0.077**	**0.582-0.886**	**<0.001**
	Age	-0.024	-0.021	0.118	-0.258-0.210	0.840
	Sex	-0.150	-0.005	2.084	-4.285-3.985	0.943
	Years of education	-0.321	-0.103	0.232	-0.782-0.140	0.170
	Duration of illness	-0.064	-0.047	0.127	-0.316-0.189	0.617
	Total number of episodes	-0.015	-0.016	0.071	-0.157-0.127	0.831
	HAM-D score	-0.109	-0.021	0.357	-0.817-0.599	0.761
	YMRS score	**1.564**	**0.170**	**0.649**	**0.278-2.851**	**0.018**
	Smoking status	0.089	0.003	1.852	-3.584-3.763	0.962

B, unstandardized coefficient; β, standardized coefficient; SE, standard error. Variables were entered hierarchically (block-wise). Model 1: ASRS total score. Model 2: Model 1 + age, sex, and years of education. Model 3: Model 2 + duration of illness, total number of episodes, Hamilton Depression score, Young Mania score, and smoking status. Sex: 0=Male, 1=Female; smoking status: 0=No, 1=Yes. Model 1: R^2^=0.534, p<0.001; Model 2: R^2^=0.546, ΔR^2^=0.012, p=0.419; Model 3: R^2^=0.573, ΔR^2^=0.026, p=0.301. Variance inflation factors in the final model ranged from 1.04 to 2.58. Bold values indicate statistically significant associations (p<0.05).

In the second regression analysis examining the effects of ASRS subscales on BIS-11 total score (**Table 6**), both subscales were significant independent predictors when entered together in Model 1, explaining 53.7% of the variance (R^2^=0.537; p<0.001). Adding demographic variables (Model 2) did not significantly increase explained variance (ΔR^2^=0.015; p=0.331), nor did adding clinical variables (Model 3), which raised R^2^ to 0.576 (ΔR^2^=0.025; p=0.323). In the final model, both the ASRS Inattention subscale (B = 0.589; β=0.317; p<0.001) and the ASRS Hyperactivity-Impulsivity subscale (B = 0.891; β=0.436; p<0.001) remained independent predictors of BIS-11 total score, with Hyperactivity-Impulsivity showing the larger standardized effect. The Young Mania Rating Scale score was again an independent, positive predictor (B = 1.516; β=0.165; p=0.022). Variance inflation factors in the final model ranged from 1.04 to 2.61, indicating no problematic multicollinearity.

**Table 6:** Multivariable linear regression analysis of the effects of ASRS subscales on BIS-11 total score.

Model	Variable	B	β	SE	95% CI	p
1	ASRS Inattention	**0.699**	**0.375**	**0.153**	**0.395-1.003**	**<0.001**
	ASRS Hyperactivity-Impulsivity	**0.901**	**0.441**	**0.168**	**0.568-1.235**	**<0.001**
2	ASRS Inattention	**0.624**	**0.335**	**0.162**	**0.303-0.945**	**<0.001**
	ASRS Hyperactivity-Impulsivity	**0.945**	**0.462**	**0.176**	**0.596-1.293**	**<0.001**
	Age	-0.107	-0.095	0.083	-0.272-0.058	0.201
	Sex	0.287	0.011	2.046	-3.770-4.344	0.889
	Years of education	-0.342	-0.110	0.227	-0.791-0.107	0.134
3	ASRS Inattention	**0.589**	**0.317**	**0.172**	**0.248-0.930**	**<0.001**
	ASRS Hyperactivity-Impulsivity	**0.891**	**0.436**	**0.183**	**0.528-1.254**	**<0.001**
	Age	-0.013	-0.011	0.119	-0.248-0.223	0.915
	Sex	0.002	<0.001	2.092	-4.148-4.152	0.999
	Years of education	-0.373	-0.120	0.239	-0.846-0.101	0.122
	Duration of illness	-0.072	-0.053	0.128	-0.325-0.182	0.576
	Total number of episodes	-0.027	-0.029	0.073	-0.171-0.117	0.708
	HAM-D score	-0.045	-0.009	0.364	-0.766-0.676	0.902
	YMRS score	**1.516**	**0.165**	**0.651**	**0.225-2.808**	**0.022**
	Smoking status	-0.158	-0.006	1.871	-3.871-3.555	0.933

B, unstandardized coefficient; β, standardized coefficient; SE, standard error; CI, Confidence interval; R^2^, Coefficient of determination; ΔR^2^, Change in variance explained compared to the previous model. Variables were included in the model using a hierarchical (block) method. Model 1: ASRS Inattention and Hyperactivity-Impulsivity subscales. Model 2: Model 1 + age, sex, and years of education. Model 3: Model 2 + duration of illness, total number of episodes, Hamilton Depression score, Young Mania score, and smoking status. Sex: 0=Male, 1=Female; smoking status: 0=No, 1=Yes. Model 1: R^2^=0.537, p<0.001; Model 2: R^2^=0.552, ΔR^2^=0.015, p=0.331; Model 3: R^2^=0.576, ΔR^2^=0.025, p=0.323. Variance inflation factors in the final model ranged from 1.04 to 2.61. Bold values indicate statistically significant associations (p<0.05).

To address the possibility that the association between ASRS and BIS-11 scores partly reflects shared item content rather than a purely empirical relationship, a sensitivity analysis was performed after removing conceptually and behaviorally overlapping items from both instruments (ASRS items 1, 2, 4, 5, 8, 11, 12, 15, 16, 17, and 18; BIS-11 items 1, 5, 6, 7, 9, 11, 14, 17, 19, and 28). These reduced scores were used only for this sensitivity analysis and are not proposed as validated alternative subscale scores. The reduced ASRS and BIS-11 total scores remained strongly and significantly correlated (ρ=0.605; p<0.001). In multivariable linear regression adjusted for age, sex, years of education, duration of illness, total number of episodes, Hamilton Depression score, Young Mania score, and smoking status, the reduced ASRS total score remained a positive, independent predictor of the reduced BIS-11 total score (B = 0.984; β=0.627; p<0.001; model R^2^=0.458, adjusted R^2^=0.409, F = 9.476, p<0.001). Results of this sensitivity analysis are presented in **Table 7**.

**Table 7:** Sensitivity analysis of the association between reduced ASRS and reduced BIS-11 total scores after removal of overlapping items.

Analysis	B	β	SE	95% CI	t	p
Reduced ASRS total score	**0.984**	**0.627**	**0.125**	**0.737-1.231**	**7.896**	**<0.001**

B, unstandardized coefficient; β, standardized coefficient; SE, standard error; CI, confidence interval. The multivariable model was adjusted for age, sex, years of education, duration of illness, total number of episodes, Hamilton Depression score, Young Mania score, and smoking status. Model R^2^=0.458, adjusted R^2^=0.409, F = 9.476, p<0.001. Bold values indicate statistically significant associations (p<0.05).

The sensitivity of the main regression model to influential observations was evaluated using Cook’s distance; eight observations with Cook’s D >4/n were identified and excluded, and the analysis was repeated. Both models were adjusted for age, sex, years of education, duration of illness, total number of episodes, Hamilton Depression score, Young Mania score, and smoking status. The positive, independent association between ASRS total score and BIS-11 total score observed in the main model (n=111; B = 0.734; β=0.676; p<0.001) persisted at a similar magnitude after excluding the eight influential observations (n=103; B = 0.718; β=0.683; p<0.001), as shown in **Table 8**.

**Table 8:** Sensitivity analysis of the association between ASRS total score and BIS-11 total score after exclusion of influential observations.

Analysis	n	B	β	95% CI	p
Main multivariable model	111	**0.734**	**0.676**	**0.582-0.886**	**<0.001**
After exclusion of influential observations	103	**0.718**	**0.683**	**0.581-0.856**	**<0.001**

Influential observations were identified using Cook’s distance (>4/n). Both models were adjusted for age, sex, years of education, duration of illness, total number of episodes, Hamilton Depression score, Young Mania score, and smoking status. B, unstandardized coefficient; β: standardized coefficient; CI, confidence interval. Bold values indicate statistically significant associations (p<0.05).

To evaluate the possible influence of treatment characteristics on the main association, treatment regimen (monotherapy vs. combination therapy) and duration of medication use were added to the main multivariable model, which was also adjusted for age, sex, years of education, duration of illness, total number of episodes, Hamilton Depression score, Young Mania score, and smoking status. Combination therapy comprised patients receiving a mood stabilizer plus antipsychotic, mood stabilizer plus antidepressant, antipsychotic plus antidepressant, or mood stabilizer plus antipsychotic plus antidepressant; monotherapy comprised patients receiving only a mood stabilizer or only an antipsychotic. After the addition of these variables, the positive, independent association between ASRS total score and BIS-11 total score persisted (β=0.704; p<0.001); neither treatment regimen (p=0.171) nor duration of medication use (p=0.170) was independently associated with BIS-11 total score (**Table 9**).

**Table 9:** Sensitivity analysis of the association between ASRS total score and BIS-11 total score after adjustment for treatment characteristics.

Variable	B	β	SE	95% CI	t	p
ASRS total score	**0.764**	**0.704**	**0.077**	**0.611-0.917**	**9.912**	**<0.001**
Combination therapy	-3.460	-0.092	2.511	-8.442-1.522	-1.378	0.171
Duration of medication use	0.365	0.098	0.264	-0.159-0.889	1.381	0.170

B, unstandardized coefficient; β, standardized coefficient; SE, standard error; CI, confidence interval. Model also adjusted for age, sex, years of education, duration of illness, total number of episodes, Hamilton Depression score, Young Mania score, and smoking status. Bold values indicate statistically significant associations (p<0.05).

Because the Young Mania Rating Scale score was, by design, close to a constant in this euthymic sample (median 0, IQR 0–0.5; 74.8% of patients scored 0), we specifically examined whether its independent association with BIS-11 total score in the main regression model was driven by a small number of influential observations. Two sensitivity checks were performed, both adjusted for the same covariates as the main model (ASRS total score, age, sex, years of education, duration of illness, total number of episodes, Hamilton Depression score, and smoking status). First, the model was re-estimated after excluding the eight observations with Cook’s D >4/n identified in the main sensitivity analysis (**Table 8**); rather than weakening, the YMRS association strengthened (B = 2.577; 95% CI 1.379–3.774; p<0.001), indicating that it is not an artifact of a few high-leverage cases. Second, YMRS was dichotomized (any residual manic symptoms [YMRS>0, n=28] vs. none [YMRS = 0, n=83]) to remove reliance on a linear-continuous assumption across a narrow range; patients with any residual manic symptoms scored, on average, 5.72 points higher on the BIS-11 than those with YMRS = 0, independent of covariates (95% CI 1.53–9.91; p=0.008). Both checks are summarized in **Table 10**.

**Table 10:** Sensitivity analysis of the association between YMRS score and BIS-11 total score.

Analysis	n	B	95% CI	p
Main multivariable model (YMRS continuous)	111	1.564	0.278–2.851	0.018
After exclusion of influential observations (Cook’s D >4/n)	103	2.577	1.379–3.774	<0.001
YMRS dichotomized (any residual manic symptoms vs. none)	111	5.718	1.525–9.912	0.008

B, unstandardized coefficient; CI, confidence interval. All models adjusted for ASRS total score, age, sex, years of education, duration of illness, total number of episodes, Hamilton Depression score, and smoking status. Influential observations were identified using Cook’s distance (>4/n), the same eight cases excluded in **Table 8**. Variance inflation factors in both sensitivity models ranged from 1.04 to 2.49, indicating no multicollinearity.

## Discussion

In this study, the relationship between self-reported impulsivity and self-reported adult ADHD symptoms was examined in 111 patients diagnosed with Bipolar Disorder Type I according to DSM-5 criteria who had remained euthymic for at least two months. Consistent with our original analysis, ASRS scores showed a strong and independent association with BIS-11 scores; a series of sensitivity analyses, addressing item-content overlap between the two instruments, influential observations, and treatment characteristics, indicated that this association is not attributable to any single one of these factors alone, although, as discussed below, part of the shared variance between the two self-report measures may still reflect their conceptual and item-level overlap rather than a wholly independent clinical relationship.

The mean age at illness onset (28 ± 9 years) and mean duration of illness (17 ± 10 years) are consistent with the literature indicating that BD is a chronic disorder typically emerging during young adulthood (1, 2). On the other hand, previous studies have reported that patients with BD and comorbid ADHD experience illness onset approximately four years earlier, suggesting that ADHD may function as a potential risk factor for the development of BD and underscoring the neurobiological interaction between the two disorders (10).

The lifetime suicide attempt rate of 20.7% observed in this sample is descriptively consistent with the substantial suicide risk reported in the BD literature, although the present study did not examine the association between impulsivity or ADHD symptom scores and suicidal behavior, and this rate should not be interpreted as evidence of such a link within our data. In other cohorts, Zakowicz et al. suggested that impulsivity, as measured by the BIS-11, is not by itself a strong predictor of suicide in BD; however, its clinical significance increases when combined with emotional dysregulation (22). Similarly, Kulacaoglu and Izci demonstrated significantly higher Barratt Total scores among BD patients with a history of suicide attempts, emphasizing impulsivity as an important antecedent of suicidal behavior (23). Whether a comparable relationship holds in the present sample remains an open question for future studies designed to test it directly.

The mean BIS-11 total score of 58.8 ± 13.6 remains within the normal range defined by conventional interpretive cutoffs for the instrument (scores of 52–71 regarded as within normal limits, ≥72 as clinically significant impulsivity), and only 18.9% of patients scored in the clinically significant range (24). This value should therefore not, on its own, be read as evidence of elevated impulsivity in this sample; the present study did not include a healthy control group against which such elevation could be established, a limitation discussed further below. The meta-analysis by Santana et al., which included 46 studies, reported that self-reported impulsivity in euthymic BD patients is on average substantially higher than in healthy controls, with a large effect size (25); our sample’s mean score lies toward the lower end of the range of sample means reported across the studies in that meta-analysis, so our findings are best read as compatible with, rather than a confirmation of, that broader literature. Our findings remain consistent with theoretical frameworks conceptualizing impulsivity in BD as both a state-related and trait-related phenomenon (5, 6), a question the present cross-sectional, single-group design cannot itself resolve.

The mean ASRS total score was 28.9 ± 12.5, with subscale means of 16.1 ± 7.3 for Inattention and 12.8 ± 6.6 for Hyperactivity-Impulsivity. Applying the validated ASRS Part A screening algorithm, 28.8% of participants screened positive for adult ADHD symptoms, indicating that, while the sample mean lies below commonly cited screen-positive cutoffs, a meaningful minority of patients met screening criteria. According to the meta-analysis by Schiweck et al., which included 646,766 participants, approximately 17.1% of adults with BD have comorbid ADHD (10), while another meta-analysis reported a lifetime prevalence of 18.6% (26); our ASRS Part A screening-positive proportion is numerically higher than these comorbidity estimates, which is expected given that ASRS Part A identifies symptom-level screening positivity rather than confirmed diagnostic comorbidity. Because the present study did not include a structured diagnostic interview for ADHD, ASRS scores and Part A positivity should be interpreted as screening measures rather than diagnostic indicators, and the term “ADHD symptoms” rather than “ADHD comorbidity” is used throughout to describe these findings.

The strong positive correlation observed between the ASRS total score and the BIS-11 total score (ρ=0.722; q<0.001) is noteworthy. At the subscale level, the strongest correlation was observed between the ASRS Hyperactivity-Impulsivity subscale and the BIS-11 Attention subscale (ρ=0.701; q<0.001). We also examined the intercorrelation between the two ASRS subscales themselves, which was moderate (ρ=0.600; p<0.001); this degree of intercorrelation is compatible with the two subscales measuring related but distinguishable facets of ADHD symptomatology, rather than indicating that one subscale is redundant with the other. BD and ADHD share common symptom clusters, including emotional dysregulation, impulsivity, and deficits in cognitive control (10, 11). This neurobiological overlap has been attributed to shared dopaminergic and noradrenergic dysfunction as well as dysregulation of the prefrontal cortex (27). Because the BIS-11 and the ASRS Hyperactivity-Impulsivity subscale also share conceptually and behaviorally similar item content (e.g., acting without forethought, difficulty remaining settled), part of this association could in principle be definitional rather than purely empirical; we addressed this directly in a sensitivity analysis (see **Table 7**), which showed that the association persists, at a somewhat attenuated magnitude, after the shared items are removed.

The ASRS Inattention subscale also showed a strong correlation with BIS-11 scores (ρ=0.631; q<0.001), and in the final adjusted regression model both the Inattention (β=0.317; p<0.001) and Hyperactivity-Impulsivity (β=0.436; p<0.001) subscales remained independent predictors of BIS-11 total score, with Hyperactivity-Impulsivity showing the larger standardized effect. This pattern indicates that, in this sample, the association between ADHD symptoms and impulsivity is not confined to the conceptually closest subscale, which somewhat weakens a construct-overlap account based solely on content proximity, although it does not rule out shared method variance between the two self-report instruments more broadly.

After FDR correction, the Hamilton Depression Rating Scale score was not significantly associated with any BIS-11 score (all q>0.05); given that eligibility required HAM-D ≤7, this variable was severely range-restricted in our sample (median 2, IQR 0–5), which is expected to attenuate correlation estimates and limits how substantively a null association can be interpreted. The Young Mania Rating Scale score, despite a similarly restricted range by design (median 0, IQR 0–0.5), showed significant positive correlations with BIS-11 total, Attention, and Motor scores (q ≤ 0.002), and remained an independent positive predictor of BIS-11 total score in both regression models (β=0.170 and β=0.165, respectively; p<0.05). We elected to include YMRS in the regression models in this revision, symmetrically with HAM-D, rather than excluding it on grounds of limited variability alone. Given its restricted range, we specifically tested whether this association was an artifact of a small number of influential observations: excluding the eight cases with Cook’s D >4/n strengthened rather than weakened the YMRS association (B = 2.577; 95% CI 1.379–3.774; p<0.001), and dichotomizing YMRS (any residual manic symptoms vs. none) confirmed a significant, independent difference (B = 5.718; 95% CI 1.525–9.912; p=0.008; **Table 10**). We therefore interpret this as suggesting that even subthreshold residual manic symptoms are associated with impulsivity during euthymia, a relationship that should nonetheless be confirmed in samples with broader symptom variability.

Regression analyses demonstrated that the ASRS total score alone explained 53.4% of the variance in BIS-11 scores (R^2^=0.534; p<0.001) and remained an independent predictor after adjustment for all covariates (B = 0.734; β=0.676; p<0.001; final model R^2^=0.573); variance inflation factors (1.04–2.58) indicated no problematic multicollinearity, and the association was materially unchanged after excluding eight influential observations identified by Cook’s distance (β=0.683; p<0.001) and after adjusting for treatment regimen and medication duration (β=0.704; p<0.001). In the subscale analysis, both the ASRS Hyperactivity-Impulsivity (β=0.436; p<0.001) and Inattention (β=0.317; p<0.001) subscales emerged as independent predictors. A *post-hoc* sensitivity power analysis indicated that, for a model of this size (N = 111, nine predictors), the minimum detectable overall effect size at 80% power was Cohen’s f^2^=0.153, well below the effect size actually observed, indicating that the sample was adequately powered to detect the reported associations, though smaller effects among individual covariates cannot be excluded. Likewise, the meta-analysis by Bartoli et al. demonstrated that the coexistence of BD and ADHD is associated with earlier illness onset, a more severe clinical presentation, and higher rates of substance use disorders (28).

From a neurocognitive perspective, persistent impairments in attention, executive functioning, memory, and processing speed among euthymic BD patients have been consistently confirmed at the meta-analytic level (3, 29). In a recent study conducted in Türkiye, Ilhan et al. reported more pronounced impairments in verbal memory and executive flexibility among patients with Bipolar Disorder Type I, supporting the notion that BD Type I represents a particularly vulnerable neurocognitive subtype (30). As emphasized in Parker’s review, dysfunction within the dopaminergic brain reward circuitry appears to constitute a common neurobiological substrate underlying both disorders (27).

## Limitations

The following limitations should be considered when interpreting the findings of this study:

### Cross-sectional design

The cross-sectional nature of the study precludes causal inferences regarding the relationship between BD and ADHD symptoms.

### Lack of a control group

The absence of a healthy control group limits the ability to compare the observed impulsivity and ADHD scores with those of a normative population.

### Nature of the assessment instruments

ADHD symptoms were assessed exclusively using a self-report screening instrument (ASRS), without a structured diagnostic interview, assessment of childhood-onset symptoms, cross-situational symptom presence, or functional impairment. Consequently, this study demonstrates an association between ASRS-measured ADHD symptom severity and self-reported impulsivity, and does not establish, and should not be read as establishing, a diagnosis of adult ADHD or a diagnosis of BD-ADHD comorbidity. Throughout the manuscript, findings are described in terms of ADHD symptom burden rather than ADHD comorbidity. No participant received a formal clinical diagnosis of ADHD in the context of this study.

### Measurement overlap

The BIS-11 and the ASRS Hyperactivity-Impulsivity subscale share conceptually and behaviorally similar item content. A sensitivity analysis removing the overlapping items showed that the association between the two instruments persisted, though at a somewhat attenuated magnitude (**Table 7**); this analysis mitigates, but does not eliminate, the possibility that part of the observed association reflects shared item content and common self-report method variance rather than a purely independent clinical relationship, since the item-removal procedure was a *post-hoc* sensitivity check rather than a validated alternative scoring of either instrument.

### Statistical power

The sample size was not determined based on an *a priori* power calculation. A *post-hoc* sensitivity power analysis indicated that the study had 80% power to detect an overall effect size of Cohen’s f^2^=0.153 or larger in the main regression model; the study should therefore be considered adequately powered for the principal association reported, although individual covariates with smaller effects may not have reached statistical significance.

### Control of clinical variables

A sensitivity analysis adjusting for treatment regimen (monotherapy vs. combination therapy) and duration of medication use did not alter the main association (**Table 9**); however, medication exposure was characterized only in broad categorical terms, and specific medication classes, dosages, and polypharmacy could not be fully accounted for in the statistical models.

### Limited variability

YMRS scores remained close to a constant by design (median 0, IQR 0–0.5), with 74.8% of patients scoring 0. Despite this restricted range, the association between YMRS and BIS-11 total score was robust across two targeted sensitivity checks: it strengthened rather than weakened after excluding influential observations, and it remained significant when YMRS was dichotomized (any residual manic symptoms vs. none), removing reliance on a linear-continuous assumption (**Table 10**). We therefore retained YMRS in the regression models, while noting that confirmation in samples with broader variability in manic symptoms remains warranted.

### Recruitment documentation

Although participants were recruited consecutively from among eligible attenders during the study period, the exact number of patients screened for eligibility, the number excluded and the reasons for exclusion, and the proportion of eligible patients who declined participation were not systematically logged during data collection and could not be reconstructed for this report.

## Strengths of the study

### Verification of sustained euthymia

By applying a minimum two-month remission-duration criterion (HAM-D ≤7, YMRS ≤5) rather than relying on symptom scores at a single assessment point alone, the study enabled the investigation of impulsivity as a trait characteristic independent of acute mood episodes.

### Homogeneous clinical sample

The inclusion of only patients with Bipolar Disorder Type I minimized clinical heterogeneity arising from differences among bipolar disorder subtypes.

### Hierarchical modeling approach

The use of hierarchical multivariable linear regression analyses allowed the sequential control of demographic variables (age, sex, education) and clinical variables (duration of illness, number of episodes, smoking status), thereby demonstrating the independent predictive effect of ASRS scores on impulsivity.

### Dimensional analysis

Examining ADHD symptoms not only through total scores but also at the subscale level (inattention vs. hyperactivity-impulsivity) showed that both dimensions were independently associated with impulsivity, with the hyperactivity-impulsivity dimension (β=0.436) showing the stronger standardized effect.

## Conclusion

This study demonstrates that self-reported impulsivity in euthymic individuals with Bipolar Disorder Type I is strongly and independently associated with self-reported adult ADHD symptoms, particularly the hyperactivity-impulsivity dimension, independent of demographic and clinical variables and robust across sensitivity analyses addressing item-content overlap, influential observations, and medication. Regression analyses showed that the ASRS total score alone explained just over half of the variance in BIS-11 scores and remained an independent predictor even after adjustment for all covariates (B = 0.734; β=0.676; p<0.001). These findings are consistent with contemporary meta-analytic evidence suggesting a shared dopaminergic-noradrenergic neurobiological substrate between BD and ADHD (25, 27); because ADHD symptoms were assessed only by self-report screening rather than structured diagnostic interview, they should be understood as evidence of shared symptom burden rather than confirmed diagnostic comorbidity.

These findings suggest that assessment of ADHD symptoms may warrant consideration in the routine clinical evaluation of euthymic patients with Bipolar I Disorder, though the present design did not evaluate the diagnostic accuracy or clinical benefit of such screening. Previous studies have shown that the prevalence of ADHD comorbidity in BD ranges between 17% and 19%, and that this comorbidity is associated with earlier illness onset and a more severe clinical course (10, 26, 28); whether attention to ADHD symptoms in this population also contributes to identifying patients at higher risk of suicide-related or other adverse outcomes was not tested here and remains a hypothesis for future, purpose-designed studies.

Future studies employing longitudinal designs, structured ADHD diagnostic interviews, comprehensive neuropsychological test batteries, and healthy control groups will provide a more robust understanding of the causal nature and clinical implications of the relationship between impulsivity and ADHD symptoms.

## Data Availability

The original contributions presented in the study are included in the article/supplementary material. Further inquiries can be directed to the corresponding author.
